# The role of lysosomal peptidases in glioma immune escape: underlying mechanisms and therapeutic strategies

**DOI:** 10.3389/fimmu.2023.1154146

**Published:** 2023-06-16

**Authors:** Hao Liu, Jie Peng, Linzhen Huang, Dong Ruan, Yuguang Li, Fan Yuan, Zewei Tu, Kai Huang, Xingen Zhu

**Affiliations:** ^1^ Department of Neurosurgery, The Second Affifiliated Hospital of Nanchang University, Nanchang, China; ^2^ The Second Clinical Medical College of Nanchang University, Nanchang, China; ^3^ Jiangxi Key Laboratory of Neurological Tumors and Cerebrovascular Diseases, Nanchang, China; ^4^ Institute of Neuroscience, Nanchang University, Nanchang, China; ^5^ Jiangxi Health Commission (JXHC) Key Laboratory of Neurological Medicine, Nanchang, China

**Keywords:** lysosomal peptidases, glioma, immune escape, autophagy, cell signal pathways, cathepsin

## Abstract

Glioblastoma is the most common primary malignant tumor of the central nervous system, which has the characteristics of strong invasion, frequent recurrence, and rapid progression. These characteristics are inseparable from the evasion of glioma cells from immune killing, which makes immune escape a great obstacle to the treatment of glioma, and studies have confirmed that glioma patients with immune escape tend to have poor prognosis. The lysosomal peptidase lysosome family plays an important role in the immune escape process of glioma, which mainly includes aspartic acid cathepsin, serine cathepsin, asparagine endopeptidases, and cysteine cathepsins. Among them, the cysteine cathepsin family plays a prominent role in the immune escape of glioma. Numerous studies have confirmed that glioma immune escape mediated by lysosomal peptidases has something to do with autophagy, cell signaling pathways, immune cells, cytokines, and other mechanisms, especially lysosome organization. The relationship between protease and autophagy is more complicated, and the current research is neither complete nor in-depth. Therefore, this article reviews how lysosomal peptidases mediate the immune escape of glioma through the above mechanisms and explores the possibility of lysosomal peptidases as a target of glioma immunotherapy.

## 1 Introduction

Glioma is the most common primary intracranial tumor, with an overall incidence after age-adjusted ranging from 4.67 to 5.73 per 100,000 (1). Gliomas are tumors that originate from glial cells or precursor cells (2). According to histopathological classification, gliomas include glioblastoma(GBMs), astrocytoma, oligodendroglioma, ependymoma, oligodendroglioma (mixed glioma), and some rare tissues pathology (3). The 2021 World Health Organization classification of gliomas comprises seven grades (4). For the first time, a large subset of these tumors was defined based on IDH mutations and 1p/19q co-deletion (5). Due to the early invasion of surrounding tissues, high recurrence rate, rapid progression, and inability to completely eliminate such tumors, they have not been successfully treated so far (6). Surgery, radiotherapy, and chemotherapy can only increase survival time and cannot radically cure this disease. Although immunotherapy has improved survival in patients with different types of cancer, it can easily lead to drug resistance (7). The lack of effective treatment for glioma is related to its immune escape (8).

Mechanisms leading to tumor evasion of immune attack include immune editing and the formation of an immunosuppressive environment within the tumor (9). In the tumor microenvironment (TME), tumor cells produce chemokine CCL22 to mediate the entry of regulatory T cells (Tregs) into the TME (10). Tregs release TGF-β to suppress the activity of CD8+ cytotoxic T lymphocytes (11). Thus, tumor cells evade immune monitoring. Tumors can also evade immune recognition by modulating antigen expression, MHC-I surface levels, and changes in antigen presentation and processing mechanisms in tumor cells (12). In addition, tumors can destroy CTL function by producing immunosuppressive cytokines such as TNF-α,IL-6, IL-10, and IL-1β—thereby evading immune surveillance (13). Limited cell apoptosis in tumor cells may promote cell survival and resistance to therapy by regulating the TME and generating cancer-promoting effects (14).

Autophagy is closely related to the immune response system (15). Yoshinori Ohsumi was awarded the Nobel Prize in Physiology or Medicine in 2016 for discovering the mechanism of autophagy, which has become the focus of many researchers. Autophagy is a highly conserved catabolic process that is activated under various cell stress conditions, such as hypoxia, nutritional deficiency, and chemotherapy induction. Autophagy then facilitates the degradation process of protein, lipid, nuclei, and mitochondria to prevent cell damage and react to various cytotoxic damage. According to the mode of transport of intracellular substrates to lysosomes, autophagy is a degradation process and can be classified into three main types: macroautophagy, microautophagy, and chaperon-mediated autophagy (CMA) (16). Under normal conditions, autophagy functions to promote cell survival and protect the stability of the chemical environment within the cell. Autophagy dysfunction is associated with cancer, cardiovascular disease, neurodegenerative disease, metabolic disease, infectious disease, kidney disease, lung disease, musculoskeletal disease, and ophthalmic disease (17). However, autophagy plays a dual role in glioma (18). Autophagy has been found to enhance the anti-tumor immune response in some cases, while in others it has been found to promote immune escape from tumors. In malignant tumors such as glioma (19), liver cancer (20), lung cancer (21), and breast cancer (22), autophagy has been artificially associated with tumor immunotherapy, chemotherapy, and resistance to targeted therapy (23–25). Studies have found that autophagy can regulate tumor immunity by regulating the antigen presentation process of MHC class I molecules in dendritic cells, exosomes, mitochondrial function, the PD-L1/PD-1 pathway, and the activation and differentiation of immune cells.

Lysosomes are the primary site for the degradation of endocytosed extracellular and autophagy-sequestered intracellular material (26), and normal lysosomal function requires the participation of lysosomal enzymes. In mammalian cells, most major lysosomal peptidases can be divided into four main families: Aspartate Histone (D and E), Serine Histone (A and G), Asparagine Endopeptidase, and Cysteine Histone (B, C, F, H, K, L, O, S, V, W, and X/Z) (27). Lysosomal peptidases participate in adaptive and innate immunity, toll-like receptor (TLR) signal transduction, regulation of antigen presentation, differentiation, migration, apoptosis, autophagy, cytokine secretion, and cytotoxicity (28).

The biological mechanisms of glioma immune escape hinder the implementation of efficient immune-mediated cancer elimination and are not fully understood (29). Therefore, the purpose of this review is to summarize the role of lysosomal peptidases in glioma immune escape, and provide an idea for the diagnosis and treatment of glioma.

## 2 Autophagy promotes the immune escape process

### 2.1 By regulating the PD-1/PD-L1 checkpoint pathway

Current studies have shown that the PD-1/PD-L1 pathway manipulates immune tolerance and is mediated by the TME. The PD-1/PD-L1 pathway is also associated with antitumor responses induced by tumor cytotoxic secretion and T-cell activation. PD-1 can inhibit innate immunity and adaptive immunity, and is highly expressed in T cells, NK cells, monocytes, macrophages, B lymphocytes, activated T cells, and dendritic cells. PD-L1 expression is often associated with counteracting antitumor immune responses (30). Therefore, blocking the PD-1/PD-L1 immune checkpoint is believed to sensitize tumor immunotherapy. However, the EGFR/B3GNT3 pathway inhibits the autophagic decomposition of PD-L1 and promotes immune escape in breast cancer (31); DHHC-3 can cause a similar effect by palmitoylating PD-L1 (32). In contrast, HIP1R in tumor cells can induce the autophagic degradation of PD-L1 and enhance T-cell toxicity to tumor cells (24). Targeting the autophagy-related protein Vps34 converts cold immune tumors into hot immune-infiltrating tumors and enhances anti-PD-1/PD-L1 efficacy, that is, inhibits the effect of immunotherapy and promotes tumor growth (33).

### 2.2 By regulating MHC class I/II molecules

Major histocompatibility complex (MHC) molecule I mainly present exogenous antigens that are interpreted by the proteasome, then handled by exopeptidase in the endoplasmic reticulum, and finally combined with MHC I molecules on the cell external surface and presented to CTLs (34). Downregulated expression of MHC class I molecules by tumor cells is a kind of immune-evasion mechanism of cancer (35). MHC class II molecules are mainly expressed on mature antigen-presenting cells. Compared with MHC I, the antigen peptides bound by MHC class II molecules can be more than 13 amino acids in length and accommodate peptide side chains, making the peptides it binds more diverse (36). In pancreatic ductal adenocarcinoma cells, MHC I is degraded by lysosomes through an autophagy-dependent mechanism associated with the autophagy receptor NBR1, and inhibition of autophagy restores MHC I molecular levels (37). Other studies have shown that the autophagy-related protein Atg5 is a molecule necessary for antigen phagocytosis and presentation to MHC II (38). Gemcitabine is a nucleoside analogue, has been widely used for treatment of many diseases of anti-cancer drugs, including ovarian cancer, bladder cancer, non-small cell lung cancer, pancreatic cancer and breast cancer. Gemcitabine inhibits the immune function of macrophages by reducing the synthesis of MHC class II molecules and the secretion of TNF-α and IL-6 through autophagy inhibition (39).

### 2.3 By regulating the proliferation and differentiation of various immune cells in the tumor microenvironment

Autophagy is also very important in the functional regulation of various immunoactive cells present in the TME, including tumor-associated macrophages, dendritic cells, and T cells, activation of c-Jun N-terminal Kinase (JNK), activation of ATG5 cleavage-induced autophagy, promotion of monocyte-to-macrophage differentiation, production of cytokines, and prevention of monocyte apoptosis (40). Autophagy also promotes M2 polarization of tumor-associated macrophages through the STAT-3 pathway (41). Autophagy has been proved to play an integral role in degranulation, phagocytosis, neutrophil granulopoiesis, and neutrophil extracellular traps (42). Autophagy can also regulate CD36 molecule expression in dendritic cells, and Atg5 is required for antigen phagocytosis and presentation in MHC class II molecules (38). Autophagy is also an important regulator of T-cell homeostasis, activation, metabolism, cell differentiation, and senescence (43).

### 2.4 Others

One study indicated that glioma stem cells activate autophagy through BCL2/Adenovirus E1B 19 KDa Protein-Interacting Protein (BNIP3), giving tumor cells the ability to adapt to hypoxia. In addition to adaptation to hypoxia, bip-3-mediated autophagy not only promotes the growth of GBMs but also promotes chemoresistance through the MT1-MMP-JAK-STAT axis (44). Inhibition of autophagy-related 4C cysteine peptidase (ATG4C) can arrest tumor cells in the G1 phase and promote apoptosis to inhibit the progression of glioma, indicating that autophagy may promote glioma by promoting the proliferation of tumor cell invasion (45). Chloroquine, an inhibitor of autophagy, can partially block glioma progression using chloroquine, indicating that autophagy is associated with glioma progression (46). In addition, autophagy is also a crucial predictor of the prognosis of glioma patients. The expression of autophagy-related proteins like beclin 1, p62, and autophagy-related protein light chain (LC3) are negatively associated with the prognosis of glioma patients, especially in tumors; the higher the level, the higher the expression level of these proteins (47).

Although the contents of autophagosomes can be degraded by lysosomal proteases (mainly cathepsin B, D, L, and S), lysosomal proteases can also regulate the autophagic process through some signal pathways (e.g., MAPK and ERK), which is closely related to the aggressiveness of tumors. Thus, lysosomal proteases may induce immune escape by regulating the autophagic process.

## 3 Lysosomal peptidases promote glioma immune escape

Lysosomal peptidases can promote the immune escape of glioma cells through a variety of complex mechanisms, including the regulation of autophagy, immune cell activity, apoptosis, and MHC molecules; and the stimulation of cytokine production and epithelial interstitial transformation (EMT). The regulation of these mechanisms by lysosomal peptidases is more complex, and we illustrate it from a variety of perspectives.

### 3.1 Lysosomal peptidases regulate autophagy through signaling pathways

**Figure 1:**
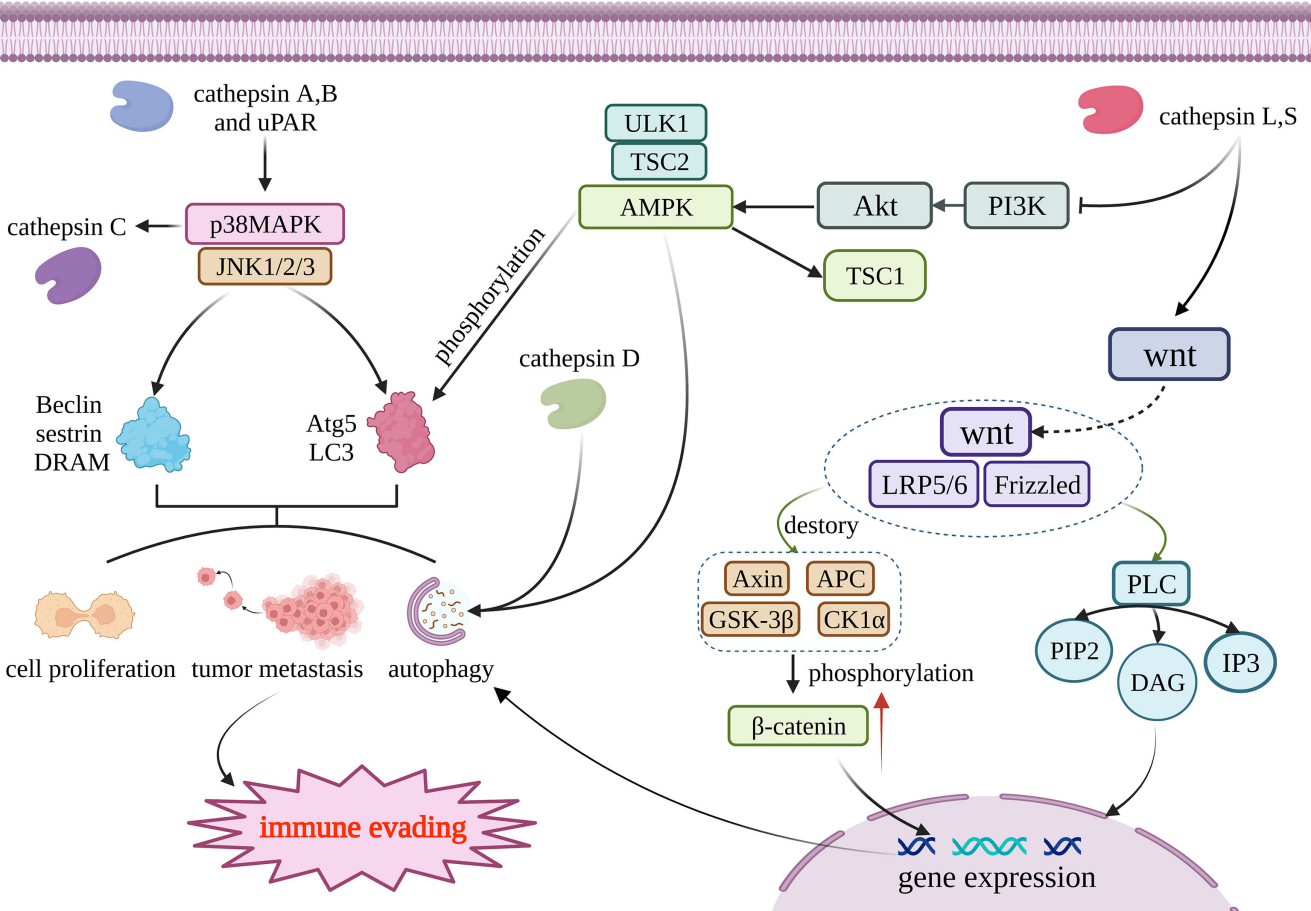
Cathepsin regulates autophagy through four pathways. Cathepsin A and B acts on MAPK/JNK pathway, and its reduction can promote the production of Beclin, sestrin, DRAM, Atg5 and LC3. Those materils promote autophagy, tumor metastasis and cell proliferation; AMPK can promote the production of Atg5 and LC3 through phosphorylation and then regulate autophagy. Cathepsin D and AMPK work together to activate autophagy; Cathepsin L and S through wnt/β- Catenin pathway, and resulting in β- Catenin accumulates and generates DAG and IP3, thus promoting gene expression to promote cell autophagy. Tissue factor acts on PI3K/AKT/mTOR pathway and regulates the generation of Atg to regulate autophagy. The above four pathways jointly affect autophagy, metastasis and proliferation of tumor cells, and participate in the immune escape process of tumors. This figure has been created with https://app.biorender.com (accessed on 19 January 2023).

The regulation of autophagy involves many complicated mechanisms, including autophagy-related proteins, autophagosome formation, and signaling pathways such as MAPK, mTOR, AMPK, and Wnt/β-catenin (48) **(** **Figure 1** **)**. Lysosomal peptidases can also impact cell autophagy involved in anti-tumor immunity through these mechanisms. Because there is only a small amount of research involving the other two mechanisms, this review mainly focuses on the signaling pathways. Lysosomal peptidases interact with these signaling pathways, which is closely correlated with the activation of cell autophagy; thus, lysosomal peptidases influence the process of tumor autophagy by relying on these mechanisms and finally regulate tumor immune evasion. According to existing studies, cathepsin S has been shown to regulate autophagy through the EGFR-ERK1 signaling pathway and play a role in tumor invasion (49) (**Figure 1**).

#### 3.1.1 MAPK/JNK signaling pathway

The MAPK signaling pathway is one of the most classic eukaryotic cell pathways. MAPK is also a highly conserved serine/threonine protein kinase with six major subfamilies: JNK1/2, ERK1/2, ERK3/4, ERK5/BMK1, ERK7/8, and p38MAPK. p38MAPK can directly regulate autophagy by directly regulating autophagy-related proteins such as LC3 and Atg5. p38MAPK can also interplay with Wnt/β-catenin by regulating the key molecule of the Wnt/β-catenin pathway, GSK3β, where Wnt/β-catenin is also a key signaling pathway of autophagy. JNK1/2/3 can regulate autophagy by the regulation of Beclin, sestrin2, and DRAM (50). The MAPK family can regulate inflammatory response, cell proliferation, cell differentiation, stress response, apoptosis, and other physiological processes (51). At the same time, it also participates in the occurrence of autophagy events induced by various stimuli.

Some studies have pointed out that autophagy activation is closely related with mitogen-activated protein kinases (MAPKs). It is worth noting that the phosphorylation of extracellular signal-regulated kinase (ERK) and c-Jun N-terminal kinase 1 (JNK1) can induce the activation of autophagy (52, 53). Caffeine therapy can weaken the transcription and translation levels of cathepsin B, reduce the activity of matrix metalloproteinase-2 (MMP-2), negatively regulate the degradation of the extracellular matrix, and reduce the invasiveness of tumors. In conclusion, caffeine impairs the invasion ability of glioma cells through the Rho-associated protein kinase–cathepsin B/FAK/ERK pathway (54). The downregulation of cathepsin B and uPAR also inhibits the MAPK/JNK pathway and impairs the migration ability of glioma cells (55). The p38MAPK inhibitor trametinib can inhibit cathepsin C expression, indicating that p38MAPK may positively regulate cathepsin C activity (56). Other studies have directly shown that inhibition of cathepsin A can restrain the p38MAPK signaling pathway and inhibit the invasion and growth of prostate cancer (57).

#### 3.1.2 AMPK signaling pathway

Amp-activated protein kinase (AMPK) is a kind of conservative serine/threonine protein kinase and plays a crucial part in various metabolic activities in cells. AMPK can also regulate autophagy by phosphorylating autophagy-related proteins, regulating mitochondrial autophagy degradation, and regulating the expression of autophagy-related genes (58). *In vivo* studies have shown that inhibition of AMPK may inhibit lysosomal peptidases activity and inhibit autophagic flux (59, 60). Knocking down STAT3 can also stimulate the TSC2-AMPKα-ULK1 signaling pathway, upregulate cathepsin D, and activate autophagy (61). Lysosomal peptidases do not appear to regulate AMPK signaling upstream but rather act in concert with AMPK to activate autophagy.

#### 3.1.3 Wnt/β-catenin signaling pathway

The Wnt pathway involves a series of life activities such as cell migration, intercellular communication, cell proliferation, embryogenesis, evolution, differentiation, and organ function maturation (62). It is also one of the important signaling pathways that regulate autophagy. It has been reported that the Wnt/beta-catenin pathway plays a part in the tumor immune control of autophagy, such as in glioma (63), hepatocellular carcinoma (HCC) (64), squamous cell carcinoma of the lung (65), and multiple myeloma (66). Furthermore, lysosomal peptidases have been shown to affect the Wnt/β-catenin pathway. One 2009 study reported that cystatin D, a cathepsin inhibitor, inhibits the growth of colon cancer cells by inhibiting c-MYC gene expression through antagonizing the Wnt/β-catenin pathway (67). This is mainly achieved by the GSK3β molecule, which affects the Wnt/β-catenin pathway. GSK3β can shuttle between the cytoplasm and the nucleus, and after entering the nucleus, GSK3β can block the transcription factors required for cancer proliferation (62). CTLD can inhibit the expression of EMT inducers SNAI1, SNAI2, ZEB1, and ZEB2 by affecting the Wnt/β-catenin pathway, and inhibit the growth of colon cancer cells. Later, in a 2012 study, in the process of hepatocellular carcinoma human mesenchymal stem cells differentiating into hepatocytes, cathepsin B and D, inorganic pyrophosphatase, phosphotriglycan isomerase, adenine phosphoribosyltransferase, lactate dehydrogenase β-chain, peptidylprolyl cis-trans isomerase A, and 11 other proteins in the Wnt pathway, the expression of hepatocytes activated by the β-catenin pathway was upregulated, indicating that cathepsin B and D are closely related to autophagy, as mediated by the Wnt/β-catenin pathway (68). The PI3K/AKT/mTOR/LC3/P62 pathway and the Wnt/Dvl/GSK3β/β-catenin pathway can interact through complex mechanisms to jointly regulate autophagy (62). Cathepsin L can also induce SNAIL expression through the Wnt/β-catenin and PI3K−AKT pathways, and regulates EMT in breast cancer and mediates tumor metastasis (69). Unfortunately, no reports about the relationship between cathepsin and Wnt/β-catenin in gliomas are available.

#### 3.1.4 PI3K/AKT/mTOR signaling pathway

The PI3K/AKT/mTOR pathway is a major regulator of cellular metabolic processes and a positive carcinogenic factor that is present in many tumors, stomach cancer, and ovarian cancer (70); it also plays a crucial role in the course of autophagy (71). Therefore, targeted inhibition of the PI3K/AKT/mTOR pathway is a new direction in anticancer therapy (72).

In a 2014 study, experimental results revealed that the use of the cathepsin S inhibitor ZFL resulted in the upregulated expression of the autophagy-related protein LC3 and the phosphorylation of AKT, mTOR, and p70S6K in human glioblastoma cells, thereby promoting autophagy and apoptosis. These results indicate that cathepsin S may inhibit autophagy and mediate immune escape through the PI3K/AKT/mTOR/p70S6K signaling pathway (73). Inhibition of cathepsin S can also inhibit TGF-β-mediated EMT through the PI3K/AKT/mTOR signaling pathway, as well as the invasive growth of glioblastoma (74). Conversely, in a study on colorectal cancer, inhibition of the PI3K/AKT pathway activated autophagy and apoptosis (75). Another study found that cathepsin D may be able to regulate the PI3K/AKT/mTOR pathway and thereby regulate autophagy (75). Moreover, impairing the activity of PI3K/AKT/mTOR can also affect the activity of lysosomal cathepsins (59), which may indicate that there is a feedback loop between lysosomal peptidases and the PI3K/AKT/mTOR pathway.

#### 3.1.5 Others

In addition to cellular pathways, lysosomal peptidases can affect autophagy through a variety of other mechanisms. Tyrosine phosphorylation of signal transducer and activator of transcription 3 (STAT3) inhibits autophagy in GBM cells (76). Bhattacharya et al. found that STAT3 knockdown enhanced LC3-II lipidation and autophagy by increasing lysosomal cathepsin D processing by activating the AMPKα/ULK1/TSC2 signaling axis (61). Their findings suggest that glioma cells are sensitized to apoptosis by inhibiting autophagy, whereas cathepsin D enhances autophagy, which may indicate that cathepsin D can mediate immune escape by activating autophagy. A number of studies have proved that cathepsin B and cathepsin L appear to be synergistic with autophagy (77, 78). Furthermore, cell death and autophagy have been closely linked to cathepsins (79). A 2012 study showed that CatD can activate autophagy and play a protective role in cell death induced by oxidative stress (80). Inhibition of cathepsin D can also improve the sensitivity of glioma radiotherapy by promoting autophagy (81). Silencing transient receptor potential mucolipin 1 (TRPML1) induces nitric oxide production, defective autophagy, and cathepsin B-dependent apoptosis in glioma cells (81). In addition to lysosomal peptidases affecting autophagy, autophagy can also in turn affect lysosomal peptidases, and the hyperactivation of autophagy by pimozide and loperamide treatment promotes LMP and lysosomal stress, thus enhancing cathepsin B activity (23).

### 3.2 Lysosomal peptidases regulate tumor microenvironment and immune cells

The TME refers to the cellular environment that cancer stem cells and tumor cells rely on to survive, which has the function of promoting cancer growth and invasion. The TME mainly comprises cancer cells, immune cells [T lymphocytes, B lymphocytes, tumor-associated macrophages (TAMs), myeloid-derived suppressor cells (MDSCs)], and cancer-associated fibroblasts. The TME includes tumor blood vessels, the extracellular matrix of the lymphatic system, and a large number of cytokines (82, 83). Glioma stem cells and other cells that exist in the glioma microenvironment play a crucial role in mediating glioma immune escape, tumor invasion, and recurrence (84). Therefore, the TME is considered an important mechanism of glioma immune escape and an important target for the treatment of glioma (85, 86). It has been found that the TME can be modulated to improve the efficacy of glioma treatment through various ways, such as regulating tumor-associated macrophages, tumor-infiltrating centriocytes, regulating signaling pathways, and cytokines. Furthermore, multiple studies have demonstrated the exciting significance of the TME in glioma therapy. A recent study involved developing a brain-targeted liposome and disulfiram/copper synergistic delivery system (CDX-LIPO) to remodel tumor metabolism and tumor immune microenvironment(TIME) by modulating the mTOR pathway, and honokiol can interact with disulfiram/copper to produce a synergistic effect for the combined treatment of GBM (87). Another study suggested that targeting neutrophil extracellular trap formation may also be a therapeutic approach to inhibit glioma (88). Furthermore, Wnt-induced signaling protein 1 (WISP1) plays a vital role in the maintenance of TAMs and glioma stem cells, suggesting that targeting Wnt/β-catenin-WISP1 signaling may improve survival and treatment outcomes in GBM patients (89).

#### 3.2.1 Tumor-associated immune cells

Tumor-associated immune cells are a vital constituent of the glioma TME and are closely linked to glioma progression and tumor immune escape (90). Lysosomal peptidases can regulate immune cells in the TME through complex mechanisms, among which are mainly cathepsin B and cathepsin L of the cysteine cathepsin family; there are also some reports about cathepsin D of the aspartate cathepsin family.

TLRs are associated with innate and adaptive immune reaction and can promote the expression of inflammatory cytokine and the immune activity of cytotoxic T cells to enhance antitumor response (91). One study found that, in Ba/F3 cells, cathepsin B/L is an essential molecule for TLR9 response and mainly affects the recognition process of the latter. Cathepsin B/L can promote B lymphocyte proliferation and CD86 upregulation through TLR9 response (91). Lack of cathepsin H leads to the impairment of TLR3 function and disables the activation of interferon regulatory factor 3, which in turn affects the subsequent secretion of IFN-β by dendritic cells, resulting in microglia death (92). In lupus patients, overexpression of cathepsin S can upregulate TLR7 and IFN-α, and promote the proliferation of monocytes and neutrophils. Whether this effect also exists in glioma patients has not been reported in the literature. TLR signaling can also regulate the activity of cathepsins B, L, and S in macrophages through positive feedback (93).

**Figure 2:**
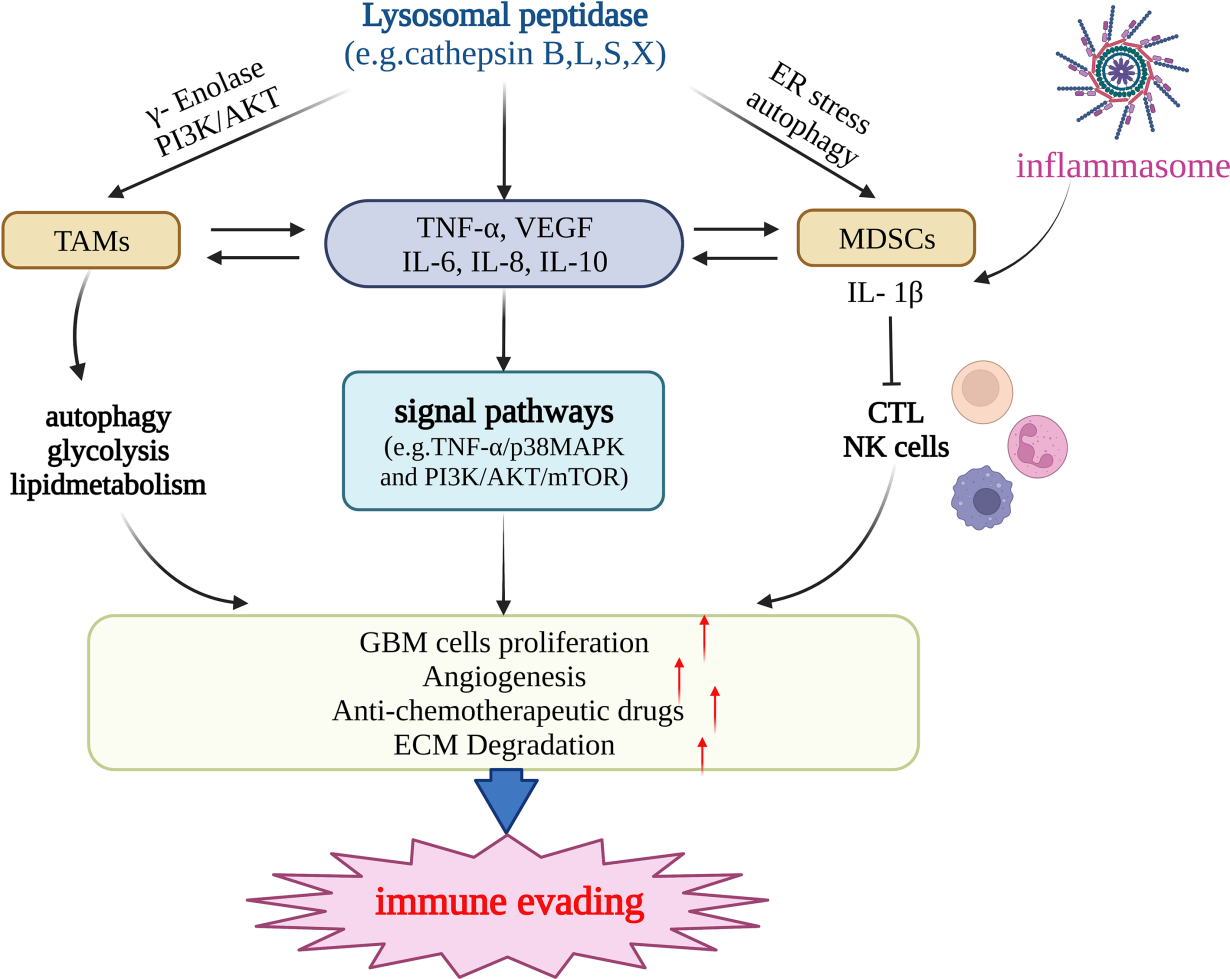
Lysosomal peptidases mediate immune evading primarily by regulating TAMs, MDSCs, and cytokines (e.g.TNF-α,VEGF,IL-6,IL-10 etc.). Lysosomal peptidases stimulates TAMs through gamma-enolase and PI3K/AKT signaling pathway to activate autophagy and regulate glycolysis and lipid metabolism, stimulates immune cells to produce cytokines such as TNF-α, VEGF,IL-6 and IL-10. On the one hand, these cytokines act on TAMs and MDSCs and regulate their function; on the other hand, they activate the signaling pathways TNF-α/p38MAPK and PI3K/AKT/mTOR. lysosomal peptidasesto stimulate the production of IL1-β by MDSCs and inhibit the function of cathepsin L and NK cells. The inflammasome also has this effect. The above three pathways jointly promote the proliferation of glioma cells, the formation of tumor neovascularization and the degradation of extracellular matrix. Eventually, glioma invasion and metastasis occur and immune escape occurs. This figure has been created with https://app.biorender.com (accessed on 19 January 2023).

Recent studies have suggested that cathepsin can mediate immune evasion by regulating tumor-associated myeloid cells and MDSCs (**Figure 2**). Tumor-associated myeloid cells mainly include TAMs and MDSCs (94). TAMs, the main culprit in promoting immune escape from the TME, are a crucial factor in the progress of treatment resistibility and are often associated with poor prognosis. With both M1-pro-inflammatory and M2-anti-inflammatory phenotypes, in malignancies, macrophages tend to transition to the M2 phenotype (83). High levels of γ-enolase, which plays a significant regulatory role in cell proliferation and aerobic glycolysis, are correlated with the proliferation, aerobic glycolysis, and immune escape of tumor cells (95). Cathepsin X is upregulated in glioma cells and promotes the proliferation of TAMs by acting on γ-enolase to activate the PI3K/AKT signaling pathway, supports tumor growth, improves GBM cell viability, and promotes GBM-related immune cell proliferation (96). One study showed that TAMs were a crucial constituent of the TME of pancreatic cancer. Cathepsin B, L, and S can induce a series of effects in tumor cells by affecting autophagy, including the enhancement of PGE2 synthesis, glycolysis, and lipid metabolism, and by guiding the transformation of the M2 phenotype of tumor-related macrophages, promoting angiogenesis and tissue remodeling, inhibiting anti-tumor immunity, and mediating immune escape (97). MDSCs mainly include two major cell populations, granulocytes or polymorphonuclear cells and monocytes, and are a predictive marker of cancer (98). MDSCs can inhibit the function of cytotoxic T cells (CTLs) and natural killer (NK) cells and promote tumor immune escape (99). Cysteine cathepsin is highly expressed in the MDSCs of highly metastatic breast cancer. Therefore, inhibiting cysteine protease can reduce the occurrence of breast cancer-related osteoclasts, as well as metastatic bone disease (100). LCL521, a lysosomotropic inhibitor of acid ceramidase, can target cathepsin B/D, leading to MDSC death by terminating autophagy and endoplasmic reticulum stress (99). Therefore, it can enhance the immune function of CTL, which is beneficial for CTL-based tumor immunotherapy. MDSCs can induce CD4+ T cells to secrete IL-17 by producing IL-1β, which is resistant to the anticancer effect of chemotherapeutic drugs, whereas IL-1β depends on cathepsin B to activate the NLRP3 inflammasome (101). Using Tick Cysteine Protease Inhibitor RHcyst-1, a cathepsin inhibitor and a member of the cystatin 1 family, can decrease and increase the activity of MDSCs in peripheral blood mononuclear cells (PBMC) and the spleen, respectively (102). In breast cancer, cathepsin L/X can also increase the activity of MDSCs in breast cancer and are related to breast cancer invasion (103). Cathepsin B also promotes the formation of neutrophil extracellular traps and modulates tumor aggressiveness *in vitro* (104). In summary, lysosomal peptidases are more likely to promote the immune escape process of tumors by enhancing the activity of MDSCs (**Figure 2**).

#### 3.2.2 Integrin

Integrins can bind to the extracellular matrix, participate in the actin network after binding to the actin cytoskeleton, activate the Ras-ERK, PI3K/AKT, and YAP/TAZ pathways, and activate intracellular signals to regulate complex cellular biology learned behaviors, including immune escape, survival, proliferation, and migration (105). Integrin signaling can initiate a variety of stem cell functions and mediate cell adhesion. Deregulation of integrin signaling in tumors enhances the immune evasion and invasiveness of tumor cells (106). In a study on breast cancer, inhibition of cathepsin K restrained the adhesion, migration, and metastasis of cancer cells through integrin molecules and their downstream targets: MMP-9, PI3K, and the MAPK signaling pathway (107). Studies have found that cathepsin X activates macrophage antigen-1 receptor–dependent phagocytosis and cell adhesion by interacting with the integrin receptor β2 subunit, as macrophage antigen-1 receptor is an inhibitor of lymphocyte proliferation. Conversely, the regulation of LFA-1 by cathepsin X promotes lymphocyte proliferation (108), which suggests that cathepsin X may have dual effects on lymphocyte activation through integrin receptors.

#### 3.2.3 NK cells

NK cells and CTLs are important components of the body’s cellular immune response; they can induce apoptosis mainly through the death receptor pathway and the extracellular release of granzymes. They also play an indispensable role in immunity against pathogens and tumor cells (109). Progranzymes must be processed in lysosomes to be converted into active granzymes (110). Among them, cathepsins C, H play an important role. Cystatin F produced in immune cells and tumor cells can be internalized into NK cells to inhibit the activity of cathepsins C, H, and L, reduce the cytotoxicity of NK cells, and play a part in facilitating cancer cell metastasis (111).

Previous works have shown that the suppression of cathepsin X affects the immune function of dendritic cells and T cells (112), but whether this affects NK cells is not clear. Interestingly, a recent study showed that cathepsin X has no effect on the adhesion between Jurkat T cells and target cells. In this study, cathepsin X did not affect the formation of immune synapses in NK cells but entered into target cells with the release of cytotoxic particles. Therefore, the inhibition of cathepsin X does not impair NK cell cytotoxicity (113).

#### 3.2.4 Tregs

Tregs are a type of immunosuppressive cells that also have a significant role in the immune escape of glioma (86). One study found that cathepsin S inhibits Treg immunosuppressive activity, which reduces T cell immunity under normal conditions but enhances the immune killing of CD8+ T cells in bladder cancer cells (114). Similar mechanisms may exist in gliomas, but research is still needed to confirm them.

### 3.3 Lysosomal peptidases and cytokines

In the TME, cytokines in the background can also cause chronic inflammation and tumor immune escape. Common cytokines include TNF-α, IL-6, IL-8, and IL-10, which have the effect of promoting tumor invasion, growth, and angiogenesis (83). Nowadays, numerous studies have revealed that lysosomal peptidases can regulate the synthesis of cytokines, thereby affecting the TME and immune cells, as well as participate in tumor immune escape. For example, cathepsin C produced by tumor cells participates in the recruitment of neutrophils and the formation of extracellular traps by promoting the processing and activation of IL-1β and NF-κB, upregulating IL-6 and the chemokine CCL3, and promoting breast cancer progression and lung metastases (115). Cathepsin K in rectal cancer cells stimulates the synthesis of cytokines such as IL10 and IL-17 and promotes the metastasis of colorectal cancer cells through the NF-κB pathway (116).

#### 3.3.1 TNF-α

Tumor necrosis factor-α (TNF-α) is a classic cytokine that can be found in inflammatory reactions, autoimmune diseases, tumor immunity, blood diseases, and even acute respiratory distress syndrome. However, as its name suggests, TNF-α should downregulate the proliferation of tumor cells and kill them. In fact, TNFs can promote tumor invasion by inducing the upregulation of chemokines and matrix metalloproteinases, and it cooperates with TGF-β to promote tumor metastasis (117). Glioma-related studies have shown that lysosomal peptidases are not regulated upstream of TNF-α, and, in gliomas, TNF-α activates cathepsin B/D to mediate cell necrosis, causing RIP1 and RIP3 kinases–driven necroptosis (118). In hepatocellular carcinoma, on the one hand, TNF-α can activate cathepsin C, and, on the other hand, cathepsin C can also activate the TNF-α/p38MAPK pathway, which has a close relationship with the growth of hepatocellular carcinoma (56). Inhibition of cathepsin K may inhibit breast cancer bone metastasis by reducing the expressions of TNF-α, IL-6, and IL-1β (119).

#### 3.3.2 TGF-β

Transforming growth factor (TGF)-β plays a vital role in the progression of glioblastoma. TGF-β inhibits tumor growth in the early stages of tumors but promotes the growth and invasion of advanced tumors (120). TGF-β can induce EMT, and play a role in promoting tumor immune escape through Smad-dependent or Smad-independent signaling pathways such as PI3K/AKT, P38 kinase, and Ras/ERK (121). Inhibition of cathepsin S reverses TGF-β-mediated EMT by inhibiting the PI3K/AKT/mTOR pathway. Inhibition of cathepsin S can also increase the expression of E-cadherin, reduce the expression of Vimentin and N-cadherin, and restore TGF-β–induced changes in cell morphology (74). Inhibition of cathepsin L can also inhibit the EMT process of breast cancer cells mediated by TGF-β through Wnt signaling and PI3K-AKT signaling pathway–related Snail (69).

#### 3.3.3 Urokinase-type plasminogen activator

The urokinase-type plasminogen activator (uPA) system is essential for endothelial cell invasion and migration during tumor angiogenesis. In cancer cells, proteolysis of the uPA system releases β-FGF and VEGF (122). An older study found that cathepsin B can hydrolyze pro-uPA to generate active double-stranded uPA (123). Inhibition of uPAR and cathepsin B can inhibit the angiogenesis around tumor cells by reducing JAK/STAT-dependent VEGF expression and impairing tumor invasion ability (124). A study on gliomas found that tivozanib, a pan-inhibitor of VEGF receptor, inhibits cathepsin B/uPA/MMP-2. The proteolytic cascade inhibits GBM cell invasion (125), which indicates that cathepsin B may be able to regulate the expression of VEGF by regulating the uPA system, and affect the angiogenesis of glioma.

#### 3.3.4 Vascular endothelial growth factor

VEGF plays an important role in the invasion and growth of various malignant tumors, including gliomas (126), which is mainly due to its ability to promote tumor angiogenesis. Therefore, glioma therapy targeting VEGF is also a current research hotspot. The VEGF-targeted therapeutic bevacizumab is currently approved for the treatment of recurrent glioma (127). Both cathepsin B and urokinase-type plasminogen-activated receptor (uPAR) are overexpressed in tumor angiogenesis, and their inhibition can inhibit tumor cell–induced endothelial cell migration by disrupting the JAK/STAT pathway, synthesis of VEGF receptor-2, cyclin D1 and cyclin-dependent kinase, inhibits tumor angiogenesis (124). *In vitro* glioma experiments showed that the inhibition of cathepsin B resulted in the downregulation of MMP-9 and VEGF expression. This study indicated that cathepsin B may be able to regulate the release of MMP-9 and VEGF, suggesting that the inhibition of cathepsin B will also inhibit tumor growth by inhibiting cathepsin B. Angiogenesis has emerged as a therapeutic approach to inhibiting glioma (128). In addition, the knockdown of VEGF receptor 2, AKT3, and PI3KCA in glioma cells by antisense RNA can significantly reduce the expression of cathepsin D, suggesting that the relationship between lysosomal peptidases and VEGF may not be unidirectional (129). This was also demonstrated in another study, where VEGF receptor inhibitors impaired the cathepsin B/uPA/MMP-2 pathway and inhibited glioma invasion (125).

In addition, in some other diseases, cathepsins can also affect cytokines, thereby affecting immune function. In neurons and glial cells in hypothermic brain injury, cathepsin C intensifies neuroinflammation by promoting the increased expression of chemokines CCL2, CXCL2, TNF-α, L-1β, IL-6, and iNOS (130). After infection by Harvey bacteria, cathepsin C can significantly enhance the body’s ability to resist *Vibrio harveii* and promote the expression of TNF-α, interferon-γ, interleukin-1β (IL-1β), IL-6, and IL-8 (131).

### 3.4 Lysosomal peptidases and apoptosis

Induction of apoptosis is one of the important mechanisms of glioma immune escape (86). Lysosomal peptidases affect apoptosis mainly by activating caspase-3, -7, -9, Bax and regulating the activity of MMP-9, and cathepsin B and cathepsin L are mainly involved in these mechanisms (132–134). Another study reported that cathepsin L has an anti-apoptotic effect in the early stage of apoptosis, and high levels of cathepsin L play a protective role against apoptosis in the case of intact lysosomes (135). Furthermore, cathepsin B plays a key role in microglial conditioned culture medium (MCM)–induced apoptosis in glioma cells, and nitric oxide is likely the major glioma cytotoxic mediator in microglial conditioned culture medium (136). Silencing the expression of MMP-9 and cathepsin B can also produce a pro-apoptotic effect by inhibiting the repair of damaged DNA, downregulating DNA repair–related protein kinases ATM, RAD51, and pCHK2 and survival signaling proteins pERK and AKT (137). The histone variant H2AX is a safeguard of the cellular genome and is involved in chromatin remodeling, apoptosis, and DNA damage response. Silencing cathepsin B and uPAR can inhibit the c-Met pathway and upregulate H2AX, increasing the radiotherapy sensitivity of glioma cells (138), and the knockdown of cathepsin L has a similar effect (139). These results may point to a new direction for glioma treatment.

### 3.5 Changing the expression patterns of MHC molecules

MHC molecules, also known as human leukocyte antigens (HLAs), play an essential role in the activation of immune cells. MHC class I antigens are composed of classical genes HLA-A, B, and C, and non-classical genes HLA-E, F, G. MHC class II antigens are mainly composed of HLA-DR, DP, and DQ (86). Among them, class I molecules, as antigen-presenting molecules of CTL, are indispensable in the immune response mediated by CD8+ T cells, aiming at the presentation of endogenous antigens (140). Class II molecules are mainly presented to antigen-presenting cells for the presentation of exogenous antigens. As NK cells can kill MHC I molecules independently, tumor cells will retain part of MHC I on the cell surface to escape recognition (141). Interestingly, changes in the expression pattern of MHC molecules can affect the process of antigen presentation, which is an important mechanism of glioma immune escape (86). However, there are not many studies on the effect of lysosomal peptidases on MHC molecules in gliomas. In a mouse model, the expression level of MHC class I molecules in mouse dendritic cells lacking cathepsin G decreased, and cathepsin G may induce the expression of MHC molecules through the PAR1 signaling pathway. CatG also upregulates the surface of glioblastoma stem cells. The expression of MHC-I molecules plays a positive role in the recognition of tumor cells by CTLs (34). However, in contrast, in another study, CatG promoted the hydrolysis of MHC class I molecules, and the lack of CatG in glioma cells prevented the complete degradation of MHC class I molecules and prevented them from being attacked by NK cells (35). This phenomenon may be related to the concentration of CatG, which is known to play a part in the post-transcriptional processing of MHC class I molecules (34). A low concentration of CatG may promote the expression of MHC class I molecules, whereas a high level of CatG can completely degrade them; however, the specific mechanism remains to be confirmed. In previous studies, cathepsin B and cathepsin D were required for the degradation of MHC class II molecular invariant chains (li) in endosomes/lysosomes, suggesting that cathepsin B and D are involved in functional MHC II molecular mediated immune responses (142). New studies show that cathepsin S is also involved in the manufacture of the MHC class II chaperon-invariant chain Li (143), as already demonstrated in head and neck cancer cells (144). In other malignancies, lysosomal peptidases can also influence tumor immunity through MHC molecules. In acute myeloid leukemia, cathepsin G attacks leukemia cells in a MHC class I molecules–dependent manner (145).

## 4 Therapy

Gliomas are classified as adult diffuse gliomas, pediatric diffuse low-grade gliomas, pediatric diffuse high-grade gliomas, and localized astrocytoma (146, 147), and different types of gliomas are treated differently. In general, treatment modalities for gliomas include surgical resection, radiation therapy, and chemotherapy. However, due to the highly aggressive characteristics of malignant gliomas and the emergence of drug resistance, the long-term treatment effect of gliomas is not satisfactory (148). This article reviews the treatment of glioma mainly from the aspect of lysosomal peptidases and discusses some therapeutic drugs and the future direction of drug research. From a research perspective, we categorize these drugs into those that affect the activity of lysosomal proteases, those that affect the gene expression of lysosomal proteases, and those that affect lysosomal function. It is important to note that these categories are not completely independent, as some substances may affect multiple mechanisms of action simultaneously (**Table 1**).

**Table 1:** Therapeutic drugs or therapeutic targets for glioma.

Therapeutic drugs	Site of action	Effect	References
Curcumin	Skp2	Inhibits growth, promotes apoptosis, inhibits metastasis.	149
siRNA	CTSD gene, Cat D	Increase radiosensitivity and enhance autophagy.	81
PL and SCO	CTSC gene, Cat C	TBD	150
caffeine	Cat B and MAPK pathway	Inhibit invasion.	54
IGFBP-4	Cat B	growth inhibition, antiangiogenic.	151
Tachyplesin I	Cat A, Cat B and Cat D	Inhibit invasion.	152
Guggulsterone	Lysosomes and Cat B	Inhibit tumor proliferation, migration and invasion, enhance the effect of chemotherapy	(153–155)
Platycodin D	Lysosomes	inhibit tumor growth	156
mefloquine	Lysosomes	Inhibits tumor growth and angiogenesi	157
FIN56	Lysosomes	inhibit tumor growth	158
Sulbactam	Lysosomal sulfatase	inhibit tumor growth	159

### 4.1 Therapeutic drugs

#### 4.1.1 Affecting the activity of lysosomal proteases

##### 4.1.1.1 Curcumin

Curcumin is considered an effective drug in the treatment of many types of tumors, including gliomas. Studies have shown that in the treatment of glioma, curcumin plays a vital part in the treatment of tumors by downregulating the S-phase kinase associated protein 2 pathway in glioma cells (149). Fei et al. found that curcumin-induced cytotoxicity could be enhanced by inhibiting cathepsin L (159). In the experiment, it was found that curcumin inhibited the proliferation of tumor cells by intensifying cell cycle arrest and apoptosis, and also inhibited the metastasis of glioma cells. Notably, by inhibiting cathepsin L, the radiosensitivity of gliomas can also be increased (139, 160, 161), which provides a new idea for drug combination radiotherapy.

##### 4.1.1.2 Caffeine

Caffeine, a substance widely present in coffee and tea, has been shown to inhibit the MAPK pathway and induce apoptosis in osteosarcoma cells (162). Later studies found that caffeine also has a positive role in the treatment of gliomas. Cheng et al. found that caffeine can inhibit the cathepsin B and MAPK signaling pathways, thereby reducing the invasion of glioma cells (54). Therefore, caffeine may serve as a potential preventive drug for glioma, as well as an adjuvant treatment drug.

##### 4.1.1.3 Insulin-like growth factor-binding protein-4

Insulin-like growth factor-binding protein-4 (IGFBP-4) has been found to have anti-angiogenic functions in experiments and can attenuate the aggressiveness of gliomas, and is considered a potential candidate for glioma therapy (163). Functionally, cathepsins in lysosomal peptidases have basement membrane degradation and are involved in tumor invasion and angiogenesis. New research also found that the anti-angiogenic and anti-tumor effects of IGFBP-4 are related to lysosomal peptidases, especially cathepsin B. Moreno et al. demonstrated that IGFBP-4 was capable of inhibiting cathepsin B activity in glioma cells, and confirmed the ability of IGFBP-4 to inhibit cathepsin B activity *in vitro* (151). Remarkably, tumor growth was reduced by 60% in the experimental IGFBP-4-treated animal model.

#### 4.1.2 Affecting the gene expression of lysosomal proteases

##### 4.1.2.1 siRNA

In addition to cathepsin L being associated with the radiosensitivity of gliomas, another study also found that cathepsin D was expressed at significantly higher levels in radioresistant clones than in parental cells. The CTSD gene is responsible for encoding cathepsin D, which is widely considered to be closely correlated with the prognosis of glioma. Zheng et al. found in their experiments that knockdown of CTSD by small interfering RNA (siRNA) increased the sensitivity of glioma cells to ionizing radiation, and the autophagy level of tumor cells was enhanced (81).

##### 4.1.2.2 Piperlongumine and scopoletin

Cathepsin C, encoded by the CTSC gene, is a member of the cysteine cathepsin family and also belongs to lysosomal peptidases. Cheng et al. found that the synthesis of CTSC in glioma cells was significantly higher than that in non-cancer cells, and the protein expression was higher in high-grade gliomas (150). In their experiment, glioma cells were treated with piperlongumine and scopoletin separately, and the results showed that the synthesis of CTSC was significantly inhibited. Therefore, piperlongumine and scopoletin may have potential as drugs targeting CTSC for the treatment of glioma.

##### 4.1.2.3 Tachyplesin I

Tachyplesin I, a peptide present in the blood cells of *Tachypleus tridentatus*, has shown positive effects in some tumors, such as lung, stomach, liver cancer (164–166). Ding et al. found that Tachyplesin I also had therapeutic effects toward gliomas and demonstrated that Tachyplesin I inhibited glioma development by disrupting the plasma membrane and inducing glioma stem cell differentiation (167). Another study found that the expression levels of cathepsins (especially cathepsin A, cathepsin B, and cathepsin D) in lysosomes were significantly changed after Tachyplesin I treatment (152). Therefore, Tachyplesin I can inhibit tumor cell metastasis by downregulating cathepsin, so as to achieve the effect of treating glioma.

#### 4.1.3 Affecting lysosomal function

##### 4.1.3.1 Guggulsterone

Guggulsterone (GS), a phytosterol extracted from *Commiphora mukul*, has been shown to have anti-tumor cell proliferation effects in various tumors, such as pancreatic tumors, bladder carcinoma, and colorectal carcinoma (168–170). Remarkably, GS not only induces apoptosis in human bladder cancer cells but also inhibits lysosomal migration in bladder cancer cells, suggesting that it can suppress tumors through the lysosomal pathway (169). Some studies have found that GS is capable of inhibiting the proliferation, migration, and invasion of glioma cells (153). Yang et al. found that after treating glioma cells with GS, cell viability and invasion ability were significantly reduced, and the expressions of adhesion complex, MMP-2, MMP-9, and cathepsin B were also reduced (154). Therefore, GS may play an anti-glioma role through proteasome and lysosome degradation. In addition, GS was found to enhance the effect of temozolomide in glioma chemotherapy by downregulating the EGFR/PI3K/Akt signaling pathways and NF-кB activity (155, 171).

##### 4.1.3.2 Other drugs

In addition, some drugs have been less studied, and the processes by which they affect lysosomes and their mechanisms of action are not yet clear, but existing research has shown that they may also have potential therapeutic effects in the treatment of glioma. Glioma cell proliferation is dependent on cholesterol homeostasis, and inhibition of intracellular cholesterol transport can mediate cancer cell death (172, 173). Platycodin D has been shown to inhibit angiogenesis by targeting multiple signaling pathways, and can inhibit cancer cell invasion and metastasis by inducing apoptosis and autophagy (174). For glioma, Platycodin D promotes the uptake of LDL cholesterol by upregulating LDL receptors, leading to cholesterol accumulation in lysosomes and glioma cell death, and inhibits the growth of glioma (156).

Mefloquine is a drug widely used in the prevention and treatment of malaria, and has shown anti-tumor effects in many studies (175–178). Wan et al. found that mefloquine can destroy the integrity and function of lysosomes in glioma cells, leading to oxidative stress and lysosomal damage, and inhibiting the growth and angiogenesis of glioblastoma (157).

FIN56, a novel ferroptosis-specific inducer, has been identified to induce ferroptosis and autophagy in bladder cancer cells by increasing the degradation of GPX4 (179, 180). Ferroptosis also has an impact on glioma (181). X. Zhang et al. found that FIN56 can induce lysosomal membrane permeabilization and inhibit the growth of glioma cells both *in vitro* and *in vivo (* 158).

M.-M. Zhang et al. found that the expression of lysosomal sulfatase is related to the prognosis of glioma, and sulbactam can inhibit the cell proliferation related to lysosomal sulfatase, thereby inhibiting the growth of glioma (182). This study suggests that sulbactam may be a promising therapeutic agent for glioma.

### 4.2 The direction of drug research

Although specific drugs have not yet been discovered through current research, certain metabolic processes and substances play a crucial role in the proliferation and invasion of tumors. By regulating these cellular processes and the activity of substances, it is possible to inhibit the proliferation and metastasis of gliomas. Therefore, the metabolic processes and substances discussed in the following sections are likely to provide direction for future exploration of drugs for the treatment of gliomas.

#### 4.2.1 MEOX2-cathepsin S axis

There has been substantial evidence that lysosomal peptidases are important mediators of tumor development in a variety of tumors. McDowell et al. showed that cathepsin S plays an important role in tumor invasion, angiogenesis, and metastasis (183). In a previous study, inhibition of cathepsin B and MMP-9 genes in glioma cells by RNA interference successfully reduced tumor growth and angiogenesis and inhibited tumor cell invasion (184). Recent studies have also found that enhanced glioma aggressiveness is associated with increased expression of cysteine cathepsins B and S and downregulation of the endogenous cell adhesion molecule NCAM (185). Wang et al. found that the nuclear transcription factor Mesenchyme Homeobox 2 (MEOX2) contributes to the cell proliferation and motility of gliomas, and that cathepsin S of the cathepsin family of cysteine proteases is a downstream target of MEOX2 (186, 187). In conclusion, direct or indirect inhibition of cathepsin B activity and the MEOX2-cathepsin S axis in glioma cells may be a direction of future drug research.

#### 4.2.2 Cathepsin X

Lysosomal cysteine carboxypeptidase cathepsin X, which is mainly present in immune cells, is thought to be involved in intracellular signaling and plays an important role in tumor proliferation and invasion (188). Majc et al. found that the expression and activity of cathepsin X in human glioma tissues were significantly higher than those in low-grade gliomas and normal brain tissues (189). The viability of macrophages and microglia in glioma was successfully reduced by selective cathepsin X inhibitor. Thus,. cathepsin X is a potential therapeutic target for glioma. Studying the role of cathepsin X in signal transduction and finding drugs acting on cathepsin X may be important research directions for glioma treatment.

#### 4.2.3 Netrin-1

In a study on ovarian cancer, Y. Li et al. found that Netrin-1 promoted cell invasion and angiogenesis in ovarian cancer (190). In the study of Vásquez et al., it was found that Netrin-1 is also involved in the process of promoting the neovascularization of gliomas, thereby enhancing their invasiveness (191). Shimizu demonstrated that an important process in the mechanism of Netrin-1–induced glioma angiogenesis and increased glioma invasiveness is cathepsin B–dependent (192). Therefore, targeting Netrin-1– and cathepsin B–dependent pathways may be a new strategy for glioma therapy.

This section mainly discusses the treatment of glioma from the perspective of lysosomal peptidases of tumor cells themselves, but numerous studies have demonstrated that lysosomal peptidases can also originate from other cells in the TME, such as endothelial cells, macrophages, and T cells. Future research should focus on tumor cell–derived lysosomal peptidases and comprehensively study the role of other cell-derived lysosomal peptidases in the TME in the occurrence and development of gliomas. The results of such studies could ultimately be used in the early diagnosis, adjuvant therapy, and prognosis monitoring of glioma.

## 5 Conclusion

Lysosomal peptidases-mediated immune escape in glioma has become one of the factors causing poor prognosis of glioma patients. Its mechanisms mainly include activating autophagy, regulating immune cells, promoting the production and release of cytokines, affecting cell apoptosis and regulating MHC-mediated antigen presentation. Curcumin, Piperlongumine, Scopoletin, caffeine and other drugs targeting Lysosomal peptidases can negatively regulate the immune escape of glioma cells by inhibiting the formation or down-regulating the activity of Lysosomal peptidases, and can also increase the sensitivity of glioma to radiotherapy.

However, at present, the research on the mechanism of Lysosomal peptidases-mediated immune escape is not complete, and there are many possible mechanisms waiting for us to discover. Drug therapy targeting Lysosomal peptidases has only been confirmed by many studies to reduce the immune escape of glioma, but it is difficult to completely block it, and the potential adverse reactions of the drug, Factors such as price are huge barriers to the clinical use of these drugs. Lysosomal peptidases can promote immune escape not only in glioma, but also in other tumors. Perhaps the tumor therapy targeting Lysosomal peptidases will be one of the research hotspots.

## Author contributions

Conceptualization, XZ, KH, and ZT; writing—original draft preparation, HL and JP; writing—review and editing, HL, JP, LH, DR, and FY; figures, YL and JP. All authors contributed to the article and approved the submitted version.
